# Platelet Function During Extracorporeal Membrane Oxygenation in Adult Patients

**DOI:** 10.3389/fcvm.2019.00114

**Published:** 2019-08-08

**Authors:** Camilla Mains Balle, Anni Nørgaard Jeppesen, Steffen Christensen, Anne-Mette Hvas

**Affiliations:** ^1^Department of Clinical Biochemistry, Aarhus University Hospital, Aarhus, Denmark; ^2^Department of Anesthesiology and Intensive Care Medicine, Aarhus University Hospital, Aarhus, Denmark; ^3^Department of Clinical Medicine, Aarhus University, Aarhus, Denmark

**Keywords:** blood platelets, extracorporeal membrane oxygenation, extracorporeal life support, platelet activation, platelet aggregation, platelet function tests

## Abstract

**Objective:** Hemorrhagic and thromboembolic complications are common during support with extracorporeal membrane oxygenation (ECMO). As platelets play a pivotal role in hemostasis, we aimed to clarify how ECMO support affects platelet function.

**Methods:** We included 33 adult patients undergoing ECMO support at a tertiary ECMO referral center at Aarhus University Hospital, Denmark. Blood samples were collected on the first morning following ECMO initiation, and subsequently every morning until the 7th (±1) day. Platelet aggregation was evaluated by whole blood impedance aggregometry (Multiplate^®^ Analyzer) using adenosine diphosphate (ADPtest), arachidonic acid (ASPItest), and thrombin-receptor-agonist-peptide-6 (TRAPtest) as agonists. A new model was applied, taking platelet count into consideration in interpretation of impedance aggregometry analyses. On the 1st and 3rd day, platelet activation was assessed by flow cytometry (Navios) using collagen-related peptide, ADP, TRAP, and arachidonic acid as agonists.

**Results:** Blood samples from all 33 patients were analyzed on day 1 of ECMO support; 24 patients were still receiving ECMO and analyzed on day 3; 12 patients were analyzed on day 7 (±1). After ECMO initiation, platelet counts decreased significantly (*p* < 0.002) and remained low during ECMO support. ECMO patients demonstrated significantly reduced platelet aggregation on day 1 compared with healthy controls (all *p* < 0.001). However, when taking platelet count into consideration, platelet aggregation relative to platelet count did not differ from healthy controls. Flow cytometry analyses demonstrated impaired platelet activation in ECMO patients on day 1 compared with healthy controls (all *p* < 0.03). No substantial difference was found in platelet activation from day 1 to day 3 on ECMO support.

**Conclusions:** Employing impedance aggregometry and flow cytometry, we found both impaired platelet aggregation and decreased platelet activation on day 1 of ECMO support compared with healthy controls. However, platelet aggregation was not impaired, when interpreted relative to the low platelet counts. Furthermore, levels of bound fibrinogen, on the surface of activated platelets in ECMO patients, were higher than in healthy controls. Together, these findings suggestively oppose that platelets are universally impaired during ECMO support. No marked difference in activation from day 1 to day 3 was seen during ECMO support.

## Introduction

Extracorporeal membrane oxygenation (ECMO) is used to treat critically ill patients suffering from acute respiratory and/or cardiac failure unresponsive to conventional treatment (1). Recent years have seen major advances in the management and technology of ECMO support (2). Nevertheless, mortality rates remain as high as 38–42% in veno-venous (3, 4) and 59–71% in veno-arterial ECMO cohorts (3).

Bleeding and thromboembolic events frequently occur during ECMO support, greatly affecting patient outcomes (5, 6). Major bleeding is reported to occur in up to one third of adult ECMO patients (4, 7, 8), and reports of intracranial hemorrhage range from 5 to 20% (3, 9, 10). Thromboembolic complications are generally less common with intracranial infarction reported to occur in up to 5% (3, 7, 9) and venous thrombosis in 7–10% of adult ECMO patients (7, 11). The incidence of thromboembolic events might, however, be underestimated as many are subclinical and therefore overlooked (12). The high frequencies of bleeding and thromboembolic events during ECMO support emphasizes the clinical relevance of understanding the hemostatic changes occurring during ECMO support. As platelets play a pivotal role in hemostasis, it is of importance to clarify how ECMO support affects platelet function. Recent studies have investigated mechanisms *in vitro* that may affect platelet function during ECMO support, suggesting that platelets may become concurrently activated *and* impaired (13–15).

Only few studies have investigated platelet function during ECMO support in adult patients (16–22), of which the majority investigated platelet aggregation using whole blood impedance aggregometry (16–19). These studies reported reduced platelet aggregation during ECMO support compared with either before ECMO initiation (17, 18) or with healthy individuals (16, 19). However, none of these studies took platelet count into consideration, despite previous documentation of a strong association between platelet count and platelet aggregation, when employing impedance aggregometry (23–29). Our research group previously developed a model which estimates expected platelet aggregation at a range of platelet counts in healthy whole blood (23, 30). Thus, making it possible to take platelet count into consideration when evaluating platelet aggregation.

Flow cytometry is a method that is independent of platelet count, because it measures the characteristics of each single platelet (31, 32); a definite advantage when investigating platelet activation during ECMO support, as these patients are often thrombocytopenic.

We hypothesized that exposure to the extracorporeal circuit would result in platelet activation. Therefore, our primary aim was to investigate changes in platelet function from day 1 to day 3 on ECMO support, by measuring platelet aggregation and surface markers of platelet activation.

## Methods

The present study is a prospective single center cohort study conducted at a tertiary ECMO referral center at Aarhus University Hospital, Denmark. The study was approved as a quality assurance study by The Central Denmark Region Committees on Health and Research Ethics (enquiry 213-2016); therefore, according to Danish law, written informed consent was not required. The Danish Data Protection Agency approved the study (Ref. no. 1-16-02-712-17).

### Study Population

Inclusion criteria were: Patients aged 18 years or older presenting with severe cardiac and/or pulmonary failure requiring ECMO support. Furthermore, patients were only included if a blood sample could be obtained on day 1 of ECMO support. Exclusion criteria was VA ECMO following cardiac surgery; because surgery, post-operative bleeding, and massive platelet transfusion may have influenced platelet function analysis.

### ECMO Management

Patients were connected to the ECMO circuit by 17–23 Fr venous cannulas, and 15–21 Fr arterial cannulas. No patients were centrally cannulated. Mean initial blood flow rates in veno-arterial (VA) ECMO were 3.6 (±0.5) L/min and in veno-venous (VV) ECMO 3.8 (±0.4) L/min. Mean initial sweep flow rates in VA ECMO were 3.2 (±1.6) and in VV ECMO 4.4 (±2.0). The ECMO circuit was inspected for fibrin and clot formation by experienced ECMO specialists every 8 h. Post-oxygenation was evaluated, and the circuit exchanged at bedside, if fibrin or clots in the oxygenator was suspected of being the cause of the patient developing hypoxia.

### Anticoagulation and Transfusions

Patients received unfractionated heparin as an intravenous bolus injection of 5,000 IU at time of ECMO cannula insertion. Following initiation of ECMO, the activated partial thromboplastin time (APTT) was kept at 40–55 s in VV ECMO patients and 50–65 s in VA ECMO patients by intravenous infusion of unfractionated heparin. Heparinization was monitored by measurement of APTT at least four times daily. Antithrombin levels were kept above 0.80 × 10^3^ IU/L during ECMO support.

Platelet transfusions were administered to maintain platelet counts above 50 × 10^9^/L. In case of bleeding complications, the transfusion threshold was increased to a platelet count of 80–100 × 10^9^/L. The standard threshold for transfusion of red blood cells was a hemoglobin of 6.0 mmol/L.

### Clinical Data

Clinical data were extracted from the patient medical records. The following clinical data were collected: ECMO configuration; indication for support; comorbidities selected in accordance with the Charlson Comorbidity Index (33); Sequential Organ Failure Assessment (SOFA) Score modified to use the Richmond Agitation-Sedation Scale (34); Simplified Acute Physiological Score-III (SAPS-III) at time of admission to the Intensive Care Unit; 30-day mortality; transfusion of blood products; and daily medical therapy.

### Blood Sampling and Laboratory Analyses

The first blood sample (denominated day 1) was obtained on the first morning following ECMO initiation. A day was defined as 24 h onward from 00:00. Blood samples were then collected on days 2 and 3. Moreover, we collected samples on day 7 (±1), if the patients were still receiving ECMO support. A blood sample prior to ECMO initiation was possible to obtain only from patients, who had been hospitalized within the Central Region of Denmark prior to ECMO initiation. Thus, results from blood samples obtained prior to ECMO initiation could not be obtained from patients, who were put on ECMO support outside the Central Region of Denmark and subsequently transported to Aarhus University Hospital for further management. Furthermore, patients put on ECMO support for extracorporeal cardiopulmonary resuscitation (ECPR), did not routinely have blood samples taken prior to ECMO initiation.

Blood was drawn from arterial lines and anticoagulated in 3 ml hirundine tubes (Roche, Basel, Schwitzerland) for impedance aggregometry analyses, and in 3.5 ml sodium citrate 3.2% tubes (Terumo Europe, Leuven, Belgium) for flow cytometry analyses. Samples for platelet count, immature platelet fraction (IPF, reflecting platelet turnover), hemoglobin, and leucocytes were anticoagulated in 2 ml ethylenediaminetetra acid (EDTA) tubes (Becton Dickinson Bioscience, San Jose, CA) and estimated using an automated hematology analyzer (Sysmex XE-5000, Sysmex, Kobe, Japan). Samples for additional laboratory parameters, such as international standardized ratio (INR), APTT, and fibrinogen, were analyzed in 3.5 ml sodium citrate 3.2% tubes employing CS 5100i (Sysmex, Kobe, Japan). Samples for C-reactive protein and creatinine were collected in 1 ml lithium-heparin tubes and analyzed using Cobas 6000 (Roche, Basel, Schwitzerland).

### Platelet Function

#### Whole Blood Impedance Aggregometry

Platelet aggregation was investigated by whole blood impedance aggregometry using the Multiplate^®^ Analyzer (Roche, Basel, Schwitzerland). In short, hirudinized whole blood was diluted 1:1 with preheated (37°C) isotonic saline followed by 3 min of incubation time at 37°C. Adenosine diphosphate (6.5 μM, ADPtest), thrombin-receptor-agonist-peptide-6 (32 μM, TRAPtest), and arachidonic acid (AA) (0.5 mM, ASPItest) (all from Roche, Basel, Schwitzerland) were used as agonists. The analysis is based on the principle that platelets become sticky following activation by an agonist, resulting in aggregation and adherence onto two metal sensor wires in the Multiplate^®^ test cuvette; this increases the electrical resistance between the wires. The increase in resistance is expressed as aggregation units (AU) and plotted as an aggregation curve over time. The area under the curve (AU^*^min) indicates the agonist induced platelet aggregation capacity. Three different agonists were chosen to target different pathways of platelet activation: ADP stimulates the ADP receptors on the surface of the platelets, thereby inducing platelet activation; TRAP mimics thrombin by activating the platelets through the protease activated receptor-1 (PAR-1) on the platelet surface; and AA is converted by platelet enzymes to thromboxane A2 (TXA2), which activates the platelet.

Impedance aggregometry results were compared with data on healthy controls chosen to match ECMO patients on two ranked criteria: (a) gender; (b) age. Data on healthy controls for ADP and AA induced platelet aggregation were obtained from Rubak et al. (28) and data on TRAP induced platelet aggregation were obtained from Pedersen et al. (35).

To evaluate platelet aggregation relative to platelet count, we assessed platelet aggregation during ECMO support relative to a 95% prediction interval of platelet aggregation in healthy whole blood at various platelet counts. This method was established by Skipper et al. (23); after inducing varying degrees of thrombocytopenia in healthy whole blood, platelet aggregation was measured by whole blood impedance aggregometry (Multiplate^®^ Analyzer), and a 95% prediction interval for platelet aggregation relative to platelet count was established (30).

#### Flow Cytometry

Activated platelets release the content of their intracellular granules, leading to translocation of proteins from the granule membrane to the platelet plasma membrane. Thus, platelet activation can be measured as a change in the expression of these activation-dependent markers on the surface of the platelet. P-selectin is exposed on the platelet surface following secretion of the α-granule content and CD63 is exposed following secretion of the dense granule content. Furthermore, following activation, the platelet surface receptor glycoprotein (GP) IIb/IIIa undergoes a conformational change to the active conformation with a high affinity for fibrinogen binding. Thus, bound fibrinogen on the surface of the platelet is a marker of platelet activation. In flow cytometry, fluorescently labeled antibodies directed against these activation-dependent platelet surface markers are used to measure their expression, and thereby determine the degree of platelet activation following agonist stimulation.

Flow cytometry analyses were performed after 1 h of resting and completed within 2 h of sample collection as previously described by Rubak et al. (36). In short, 35 μl of HEPES buffer (NaCl 137 mM, KCl 2.7 mM, MgCl_2_ 1 mM, 4-(2-hydroxyethyl)-1-piper-azineethanesulfonic acid (HEPES)) was pooled with 5 μl of each antibody and 5 μl of agonist. Subsequently, 5 μl of whole blood was added, and samples incubated for exactly 10 min in the dark at room temperature. Following incubation, 2 ml of fixation buffer (0.2% paraformaldehyde in phosphate-buffered saline) was added to fix the cells prior to analysis.

The expression of activation-dependent platelet surface markers was measured using a combination of the following fluorescently labeled antibodies: CD42b-PE (Clone HIP1) (AH Diagnostics); CD62p-APC (P-selectin, Clone P-sel.KO2.3) (eBioscience, San Diego, CA); CD63-PECy7 (Clone H5c6) (Beckton Dickinson Bioscience, San Jose, CA); and anti-fibrinogen-FITC (Polyclonal chicken) (Diapensia HB, Linköping, Sweden). All fluorescently labeled antibodies were diluted in HEPES buffer to reach saturating concentrations previously determined (36).

To induce platelet activation, four different agonists were chosen to target different pathways of platelet activation: ADP, TRAP and AA (as used in the platelet aggregation analyses), together with collagen-related-peptide (COL). COL mimics collagen by binding to the GPVI receptor on the platelet surface, resulting in platelet activation. The following agonists were added (final concentrations): Collagen-related peptide (COL, 0.51 μg/ml) (University of Cambridge, UK); ADP (10.8 μM) (Sigma Aldrich, St. Louis, MO); TRAP (28.5 μM) (JPT, Berlin, Germany); and arachidonic acid (AA, 0.58 mM) (Sigma Aldrich, St. Louis, MO).

Analyses were performed employing a Navios flow cytometer (Beckman Coulter, Miami, USA) and processed in Navios Software version 1.3. Expressions of activation-dependent platelet surface markers were measured based on 10,000 platelets; reading times were prolonged for samples with severe thrombocytopenia in an attempt to assess as many platelets as possible. For selected patients with platelet counts below 15 × 10^9^/L, roughly 3,000 platelets were assessed. CD42b was used to identify the platelets followed by exclusion of platelet–platelet aggregates. The positive analysis regions (gates) were determined by negative controls. Expression of P-selectin, after addition of HEPES buffer instead of an agonist, was used to assess the level of pre-activation.

The degree of platelet activation was expressed as: (a) The percentage of positive platelets within a gate (also known as %gated); and (b) as the median fluorescence intensity (MFI) of all positive platelets within a gate. The percentage of positive platelets reflects the proportion of platelets that become activated in response to stimulation by an agonist. The MFI is a quantitative measure of the degree of expression of the activation-dependent surface markers on activated platelets.

Gates in the negative control were set to include 1.5–2.0% positive platelets for bound fibrinogen and CD63 and 0.1–0.2% positive platelets for P-selectin, as previously recommended (32, 37). When analyzing samples with platelet counts below 15 × 10^9^/l, gates were placed at the right edge corner of the peak in the negative control, even though the percentage of positive platelets thereby exceeded 2.0%. This was due to long reading times at such low platelet counts, resulting in the appearance of unspecific, highly fluorescent particles in the gates. The gating strategy is visualized in the supplementary data (Supplementary Figure 1).

Flow cytometry results were compared with data obtained from healthy controls (38) chosen to match ECMO patients on three ranked criteria: (a) gender; (b) age; and (c) level of pre-activation of the platelets.

### Statistics

Primary outcome was difference in platelet activation from day 1 to day 3 on ECMO support measured by flow cytometry. Our sample size calculation was based on data from investigation of platelet activation by flow cytometry in healthy individuals (36), as no data had been published on platelet function investigated by flow cytometry in ECMO patients. The percentage of platelets positive for CD63 after addition of TRAP was 85 with a standard deviation of 7. We expected the standard deviation to be greater in ECMO patients than in healthy individuals; therefore, we increased the standard deviation to 7.5. With an estimated minimal relevant difference (MIREDIF) of 5, a significance level (2-alpha) of 0.05 and a power (1-beta) of 0.90, we estimated that 24 patients, analyzed by flow cytometry on days 1 and 3, had to be included in the present study.

Categorical variables are reported as frequencies and percentages. Distribution of data for continuous variables was evaluated by Q-Q plots. Normally distributed data are described as mean ± standard deviation (SD); data not following a normal distribution are described as median with interquartile range (IQR). Differences between two sets of paired data were analyzed using a paired *t*-test, if data were normally distributed, and the Wilcoxon matched-pairs signed rank test, if data did not follow a normal distribution. For repeated measurements a mixed-model analysis was performed, if data were normally or lognormally distributed. Differences between unpaired data were analyzed using an unpaired *t*-test, if data were normally distributed, and the Mann-Whitney test, if data did not follow a normal distribution. All tests of significance were two-tailed; *p* < 0.05 were considered significant. Statistical analyses were performed using GraphPad Prism version 8.0 (GraphPad, Inc., San Diego, CA).

## Results

We present data from 33 patients treated with ECMO between September 2017 and April 2019; among which, 24 patients had flow cytometry performed on both day 1 and day 3.

Patient demographics and clinical characteristics are presented in Table 1. More than twice as many patients were initially treated with VA ECMO (*n* = 23) than VV ECMO (*n* = 10). Two patients had VA ECMO converted to a VV configuration during the course of ECMO support, and another patient had VA ECMO converted to a combined VA/VV configuration. Charlson Comorbidity Index indicated low comorbidity; 18 patients scored 0–1 and only two patients scored >3. A majority of the scores were primarily driven by age above 50 years. The median duration of ECMO was 5 days (IQR: 3–8 days). Two patients had considerably longer durations of support lasting 23 and 53 days. Seven patients died and 2 patients were weaned from ECMO before day 3.

**Table 1:** Demographic and clinical characteristics of 33 adult patients treated with extracorporeal membrane oxygenation (ECMO) on day 1 (*n* = 33), day 2 (*n* = 29), and day 3 (*n* = 24).

**Variables**	**Value**
Age (years)	49 (± 13)
Male gender	23 (70%)
ECMO configuration	
VV	10 (30%)
VA	23 (70%)
ECMO indication	
Pulmonary (Pulmonary infections, septic shock, aspiration and vasculitis)	13 (39%)
Cardiac (Cardiomyopathies, cardiogenic shock, epicarditis and hypothermia)	8 (24%)
ECPR (Cardiac arrest refractory to conventional CPR)	12 (36%)
Charlson's comorbidity index	1.5 (±1.3)
Duration of ECMO (days)	5 [3–8]
Duration from ECMO initiation to day 1 blood sample	16 h: 33 min (±6 h: 58 min)
SOFA score, day 1	13.2 (±2.7)
SOFA score, day 3	13.6 (±3.4)
SAPS-III score at ICU-admission^a^	72.4 (±19.3)
Mortality at ECMO weaning	13 (40%)
30–day mortality	17 (52%)
**Laboratory parameters, day 1 (reference interval)**	
Immature platelet fraction (0.016–0.126)	0.047 [0.032–0.080]
Hemoglobin, mmol/L (7.3–10.5^b^)	6.7 (±0.9)
INR (<1.2)	1.8 (±0.8)
Activated partial thromboplastin time, sec (20–29)	44 [36–59]
Fibrinogen, μmol/L (5.5–12.0)	8.5 (±4.7)
CRP, mg/L (<8)	139 (±107)
Leucocytes, 10^9^/L (3.5–10.0)	14.1 (±8.5)
Creatinine, μmol/L (45–105^b^)	193 [66]
**Medical treatment**	
Day 1	
Unfractionated heparin^c^ Antiplatelet drugs^d^ [asp (*n =* 4); can (*n =* 1); asp + can (*n =* 2); asp + can + tic (*n =* 1)]	20 (61%) 8 (24%)
Day 2	
Unfractionated heparin^c^ Antiplatelet drugs^d^ [asp (*n =* 2); asp + can (*n =* 1); tic (*n =* 1); asp + clo (*n =* 1)]	28 (97%) 5 (17%)
Day 3	
Unfractionated Heparin^c^ Antiplatelet drugs^d^ [asp (*n =* 3); asp + clo (*n =* 1)]	23 (96%) 4 (17%)

*Values are expressed as n (%), mean (± standard deviation), or median [interquartile range]*.

*ECMO, Extracorporeal membrane oxygenation; VV, Veno-venous; VA, Veno-arterial; ECPR, extracorporeal cardiopulmonary resuscitation; h, hours; min, minutes; SOFA, Sequential Organ Failure Assessment; ICU, Intensive care unit; INR, International normalized ratio; CRP, C-reactive protein; asp, aspirin; can, cangrelor; tic, ticagrelor; clo, clopidogrel*.

a *Available for 18 patients only*.

b *Combined reference interval for men and women*.

c *Active infusion at time of blood sampling*.

d *Administered within the past 24 h prior to blood sampling*.

Laboratory parameters, obtained on day 1 of ECMO support, are presented in Table 1. The median APTT indicates that patients were sufficiently anticoagulated with unfractionated heparin according to clinical standard. In total, nine patients received antiplatelet therapy during ECMO support administered within the past 24 h prior to blood sampling (Table 1).

### Platelet Count and Platelet Turnover

A platelet count prior to ECMO initiation was possible to obtain from 15 patients. Results from blood samples obtained prior to ECMO initiation could not be obtained from 10 patients, who were put on ECMO support outside the Central Region of Denmark and subsequently transported to Aarhus University Hospital for further management. Furthermore, 8 patients, put on ECMO support for extracorporeal cardiopulmonary resuscitation (ECPR), did not have blood samples taken prior to ECMO initiation.

**Figure 1:**
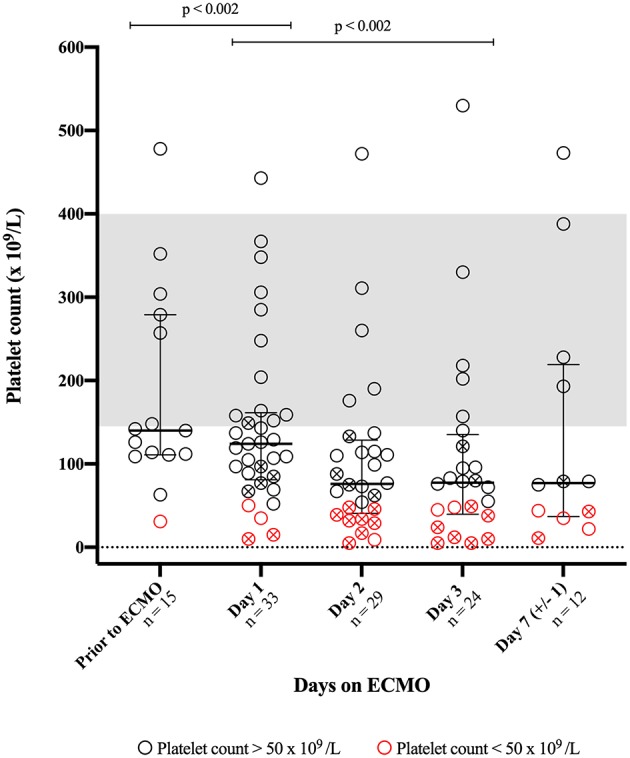
Platelet counts obtained prior to and during support with extracorporeal membrane oxygenation (ECMO). The bars indicate median and interquartile range. The gray area represents a combined reference interval on platelet count for men and women. An X-mark in the circle indicates that the patient has received platelet concentrate within 24 h prior to blood sampling.

The median duration from blood sampling prior to ECMO until ECMO initiation was 8 h 2 min (± 6 h 30 min). As seen in Figure 1, Platelet counts decreased significantly during ECMO support; from before ECMO initiation to day 1 (*p* < 0.002), and from day 1 to day 3 (*p* < 0.002). Platelet counts did not appear to change from day 3 to day 7 (± 1) (*p* = 0.74). Median platelet counts (× 10^9^/L) were 140 (prior to ECMO); 124 (day 1); 76 (day 2); 78 (day 3); and 77 (day 7 ± 1). Two patients consistently demonstrated the highest platelet counts (> 388 x 10^9^/L) throughout the course of ECMO support.

In total, 20 patients received transfusions of platelet concentrates during ECMO support, denoted by an X-mark in the circles of Figure 1; the median amount of transfused platelet concentrate per day was: 732 ml (IQR: 378–1018) on day 1; 372 ml (IQR: 368–758 ml) on day 2; 563 ml (IQR: 378–768 ml) on day 3; and 752 ml (IQR: 527–1064 ml) on day 7 (±1).

Platelet turnover accelerated during ECMO support, demonstrated by a significant increase in IPF from day 1 to day 3 of ECMO support; median IPF was 0.047 (IQR 0.032–0.080) on day 1 and 0.067 (IQR 0.045–0.133) on day 3 (*p* = 0.004).

### Platelet Function

#### Whole Blood Impedance Aggregometry

**Figure 2:**
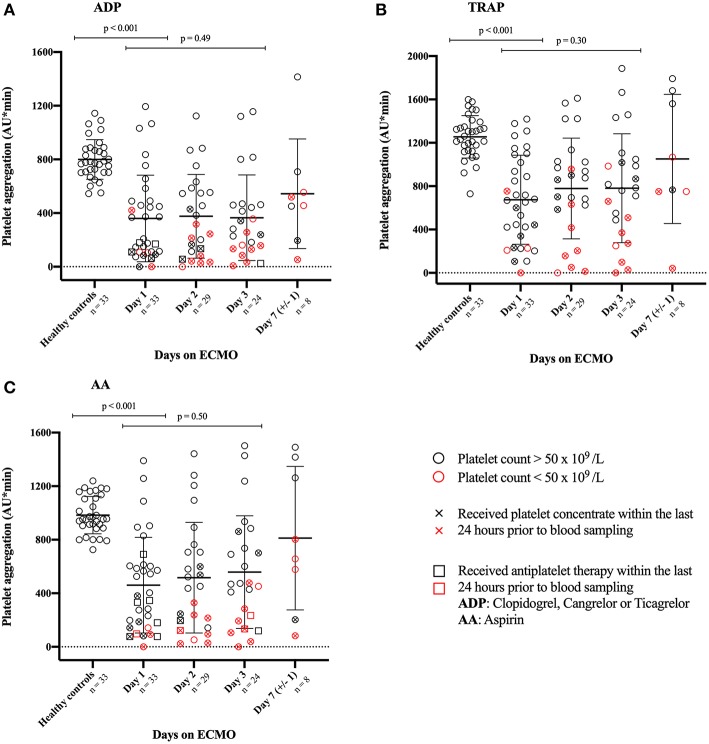
Illustrates the platelets ability to aggregate in response to stimulation by three different agonists. Platelet aggregation was measured during support with extracorporeal membrane oxygenation (ECMO) and compared with healthy controls. Agonists used to stimulate platelet aggregation: **(A)** ADP, Adenosine diphosphate; **(C)** AA, arachidonic acid; and **(B)** TRAP, thrombin receptor activating peptide-6. The bars indicate mean with standard deviation. An X-mark in the circle indicates that the patient has received platelet concentrate within 24 h prior to blood sampling. To illustrate the association between platelet aggregation and platelet count, patients with platelet counts below 50 × 10^9^ /L are marked red. Due to practical reasons, platelet aggregation on day 2 was not measured in 2 patients.

ECMO patients did not differ significantly from healthy controls on either gender or age (age: *p* > 0.86 for all agonists). As shown in Figure 2, ECMO patients demonstrated significantly reduced platelet aggregation on day 1 compared with healthy controls following stimulation by all three agonists (all *p* < 0.001). As demonstrated by the red colored symbols in Figure 2, patients with platelet counts below 50 × 10^9^/L generally exhibited the lowest levels of platelet aggregation following agonist stimulation. Figure 2 shows no significant difference in platelet aggregation from day 1 to day 3 following stimulation by any of the three agonists (ADP: *p* = 0.49; TRAP: *p* = 0.30; AA: *p* = 0.50). Overall, ECMO patients demonstrated a wide variation in platelet aggregation measured by impedance aggregometry during the course of ECMO support.

As shown in Supplementary Figure 2, the majority of ECMO patients demonstrated platelet aggregation within the 95% prediction interval on days 1, 2, and 3. Approximately one fourth of ECMO patients demonstrated platelet aggregation below the 95% prediction interval on day 1 after stimulation by ADP or TRAP. Following ADP stimulation on day 1, four patients with platelet counts above 200 × 10^9^/L demonstrated platelet aggregation below the 95% prediction interval. Of these, two patients demonstrated platelet aggregation within the 95% prediction interval on days 2 and 3. The other two patients had received antiplatelet therapy within 24 h prior to blood sampling on day 1; they were both weaned from ECMO before day 2 due to recovery (*n* = 1) or mortality (*n* = 1). The patient with the highest platelet counts (>470 × 10^9^/L) demonstrated consistently low platelet aggregation on days 2 and 3. Following TRAP stimulation on day 1, the same four patients demonstrated platelet aggregation below the prediction interval, as was seen following ADP stimulation. Two additional patients fell below the 95% prediction interval on day 1 following TRAP stimulation; of these, one patient demonstrated platelet aggregation within the 95% prediction interval on days 2 and 3; the other patient demonstrated consistently low platelet aggregation on days 2 and 3 relative to the very high platelet counts (>470 × 10^9^/L). Of note, patients with similar platelet counts demonstrated widely varying platelet aggregation throughout all days on ECMO.

#### Flow Cytometry

ECMO patients did not differ significantly from healthy controls on gender or age; the mean age was 46 (± 11) years in controls and 49 (± 13) years in ECMO patients (p = 0.34). ECMO patients exhibited a slightly higher level of pre-activation of the platelets [14% (± 8)] compared with healthy controls [10% (± 4)] (*p* = 0.03).

**Figure 3:**
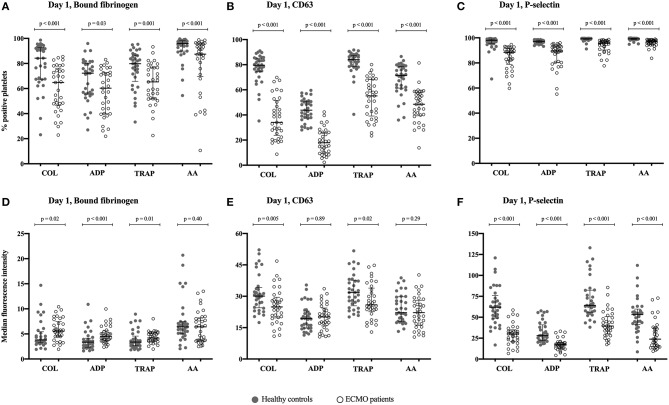
Illustrates the degree of platelet activation in response to stimulation by four different agonists. The degree of activation can be measured as a change in the expression of the activation-dependent platelet surface markers bound-fibrinogen, CD63 and P-selectin on day 1 of support with extracorporeal membrane oxygenation (ECMO). The results are compared with healthy controls, *n* = 33. **(A–C)** The percentage of activated platelets in response to stimulation by an agonist; **(D–F)** The median fluorescence intensity (MFI) of the activated platelets following stimulation by an agonist. Agonists used to activate the platelets: COL, Collagen-related peptide; ADP, adenosine diphosphate; TRAP, thrombin receptor activating peptide-6; and AA, arachidonic acid. The bars indicate median and interquartile range.

Figure 3 illustrates the percentages of positive platelets (Figures 3A–C) and MFI of the platelets (Figures 3D–F) on day 1 of ECMO support compared with healthy controls. Following stimulation by four different agonists, the percentages of positive platelets were significantly lower in ECMO patients than in healthy controls for all platelet activation markers investigated: Bound fibrinogen (all *p* < 0.03), CD63 (all *p* < 0.001), and P-selectin (all *p* < 0.001). The MFI of bound fibrinogen was significantly higher in ECMO patients than in healthy controls following stimulation by COL, ADP, and TRAP; no difference was found in MFI of bound fibrinogen following stimulation by AA (Figure 3D). Conversely, the MFI of CD63 was significantly lower in ECMO patients than in healthy controls after stimulation by COL and TRAP; whereas stimulation by ADP and AA yielded no significant difference (Figure 3E). The MFI of P-selectin was significantly lower in ECMO patients than in healthy controls for all agonists (all *p* < 0.001) (Figure 3F).

**Figure 4:**
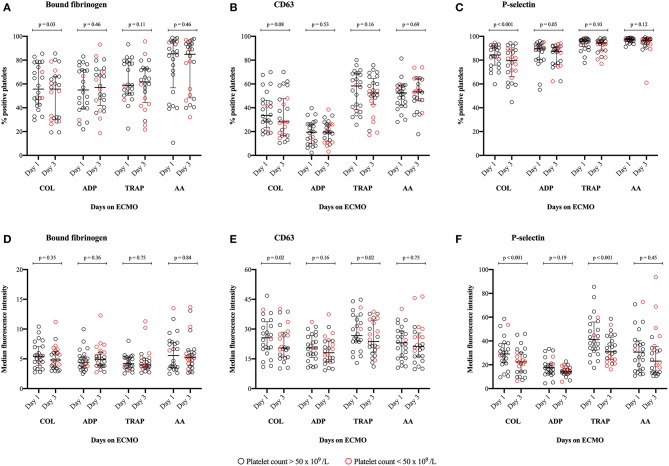
Illustrates the degree of platelet activation in response to stimulation by four different agonists. The activation can be measured as a change in the expression of the activation-dependent platelet surface markers: Bound-fibrinogen, CD63 and P-selectin. **(A–C)** The percentage of activated platelets in response to stimulation by an agonist; **(D–F)** The median fluorescence intensity (MFI) of the activated platelets following stimulation by an agonist. Platelet activation on day 1 of support with extracorporeal membrane oxygenation (ECMO) is compared with platelet activation measured on day 3 of ECMO support, *n* = 24. Patients weaned from ECMO support prior to day 3 were excluded from this analysis on the difference between day 1 and 3. Agonists used to stimulate platelet activation: COL, Collagen-related peptide; ADP, adenosine diphosphate; TRAP, thrombin receptor activating peptide-6; and AA, arachidonic acid. The bars indicate median and interquartile range.

Figure 4 illustrates the percentage of positive platelets (Figures 4A–C) and MFI (Figures 4D–F) of the activation-dependent platelet surface markers investigated on day 1 compared with day 3 of ECMO support. No significant difference was found in the percentages of positive platelets on day 1 compared with day 3 (*p* >0.05); although, there were two exceptions following stimulation with COL: Significantly reduced percentages of platelets positive for bound fibrinogen (Figure 4A: *p* = 0.03) and P-selectin (Figure 4C: *p* < 0.001) on day 3 compared with day 1. Likewise, no significant difference was found in the MFI of bound fibrinogen on activated platelets on day 1 compared with day 3 (Figure 4D: *p* > 0.35). Regarding CD63 and P-selectin, we found: Significantly reduced MFI on day 3 compared with day 1 following stimulation by COL and TRAP (*p* < 0.02), but no significant differences following stimulation by ADP and AA (*p* > 0.16) (Figures 4E,F). Overall, ECMO patients demonstrated a wide variation in expression of the activation-dependent platelet surface markers.

### Clinical Endpoints

Table 2 summarizes the use of blood products during ECMO support for all patients; 91% of patients received red blood cells (RBC); 57% received fresh-frozen plasma (FFP); and 57% received platelet concentrates during ECMO support.

**Table 2:** An overview of the use of blood products during support with extracorporeal membrane oxygenation (ECMO) for all patients (*n* = 33).

**Amount, mL**	**RBC, *n* (%)**	**FFP, *n* (%)**	**Platelets, *n* (%)**
None	3 (9%)	14 (43%)	14 (43%)
1–1,500	14 (43%)	13 (39%)	8 (24%)
1,500–3,000	9 (27%)	2 (6%)	7 (21%)
3,000–4,500	2 (6%)	1 (3%)	2 (6%)
>4,500	5 (15%)	3 (9%)	2 (6%)

*ECMO, Extracorporeal membrane oxygenation; RBC, Red blood cells; FFP, Fresh-frozen plasma; mL, milliliter*.

## Discussion

During ECMO support, platelet counts decreased despite administration of platelet concentrates. Moreover, platelet aggregation was impaired during ECMO support compared with healthy controls; this finding was challenged by the observation that platelet aggregation values relative to platelet count, in ECMO patients, were within the 95% prediction interval of platelet aggregation in healthy whole blood. Flow cytometry analyses demonstrated an impaired platelet activation in ECMO patients on day 1 compared with healthy controls. However, no substantial difference was found in platelet activation from day 1 to day 3 on ECMO support.

### Platelet Count and Turnover

Following ECMO initiation, platelet counts decreased significantly and remained low during ECMO support, despite administration of platelet concentrates to 57% of patients. This finding is consistent with previous studies reporting thrombocytopenia during ECMO support (17, 39). A consumptive loss of platelets—due to adherence to the artificial surfaces and formation of microthrombi in the circulation—has been proposed as a plausible mechanism for the decrease in platelet counts during ECMO support (40). Two recent studies found no association between duration of ECMO support and development of severe thrombocytopenia, but found thrombocytopenia to be associated with platelet count at ECMO cannulation, and the development of multi-organ failure while on ECMO (41, 42).

The significant increase in IPF from day 1 to day 3 on ECMO support indicates an augmented platelet turnover (43); this is consistent with a consumptive loss of platelets during ECMO support. Immature platelets exhibit an increased hemostatic potential (44, 45); they tend to contain more granules, and express a greater number of activation-dependent platelet surface markers compared with mature platelets (46). A high platelet turnover rate could theoretically yield a larger population of platelets that are more easily activated, thereby increasing thrombotic propensity and accelerating the consumptive platelet loss.

### Platelet Function

Compared with healthy controls, platelet aggregation was significantly lower on day 1 of ECMO support, and the platelets ability to aggregate remained low during ECMO. Patients with the lowest platelet counts generally demonstrated the lowest platelet aggregation; this finding is consistent with the well-established association between platelet count and platelet aggregation, when employing impedance aggregometry (23–29). Our impedance aggregometry findings are in line with previous studies on platelet aggregation during ECMO support in adult patients (16–19). Importantly, these previous conclusions on platelet aggregation are hampered by not considering the effect of low platelet counts on platelet aggregation. To overcome this limitation, we assessed platelet aggregation results in relation to the 95% prediction interval of platelet aggregation in healthy whole blood with varying platelet counts (30). Employing this approach, we demonstrated that platelet aggregation in ECMO patients was largely equivalent to platelet aggregation in healthy controls, when considering the impact of low platelet counts. Thus, our findings indicate that platelet aggregation during ECMO support is impaired due to a low number of platelets.

When employing flow cytometry, no association was found between platelet count and platelet activation. On day 1 of ECMO support, the platelets demonstrated a reduced ability to become activated, when stimulated by an exogenous agonist; this was demonstrated by the significantly lower percentage of activated platelets on day 1 in ECMO patients compared with healthy controls. The impaired ability to become activated could be due to vast activation of platelets in the ECMO circuit. Previous studies have suggested that platelets are affected by high shear forces in the circuit of blood-contacting medical devices, resulting in platelet activation and adherence to the surfaces of the circuit (14, 15, 47, 48). Furthermore, surface-induced platelet activation is thought to occur in the ECMO circuit, as platelets come into contact with the artificial surface of the circuit (13). Excessive activation, adhesion and aggregation of platelets may increase the risk of thrombotic complications due to the formation of microaggregates in the circulation. Thus, circulating activated platelets may be partially degranulated, circulating in an exhausted state, and therefore exhibit a decreased responsiveness to agonist stimulation, as demonstrated in the present study. Interestingly, recent studies have shown that high shear stress also causes the loss of platelet receptors (GPIbα and GPVI) important for platelet adhesion (20, 49, 50). Loss of these receptors result in decreased platelet adhesion, leading to impaired hemostatic capacity and an increased potential of bleeding.

The MFI of bound fibrinogen was significantly increased in ECMO patients compared with healthy controls for three out of four agonists. This suggests that activated platelets in ECMO patients express a larger quantity of bound fibrinogen on their surface, than healthy platelets do. This finding is consistent with studies reporting an increased expression of the activated fibrinogen receptor, GPIIb/IIIa, on platelets exposed to high shear stress, as in the ECMO circuit (51, 52). As high shear stress activates the platelets, a sequence of signaling events is triggered, resulting in the activation of the glycoprotein receptor GPIIb/IIIa, which then mediates platelet aggregation and thrombus formation (53).

On the contrary, the expression of P-selectin was consistently lower on platelets from ECMO patients than platelets from healthy controls measured by the MFI. This finding may be consistent with preceding platelet activation in the ECMO circuit and subsequent shedding of P-selectin into the circulation (54)—resulting in platelets expressing a lesser amount of P-selectin on their surface. Cheung et al. (55) measured soluble P-selectin levels in 10 neonates treated with ECMO and found significantly elevated levels of soluble P-selectin 4 h after ECMO initiation, indicating *in vivo* platelet activation. Further studies of soluble P-selectin, in adult ECMO patients, would be valuable in this regard.

Considerations on the possible clinical impact of the aforementioned findings led us to speculate, whether therapeutically increasing platelet counts in these patients, could enhance platelet aggregation; thereby reducing the risk of hemorrhage. Furthermore; increasing platelet counts may yield a larger population of platelets able to become activated, further reducing the risk of hemorrhage. Yet, a concern is the concurrent risk of thrombosis, and the possibility of vast platelet activation *in vivo* due to high shear stresses and adherence to the protein-coated surfaces of the circuit. A larger study, investigating the association between platelet count and incidence of bleeding and thromboembolic events during ECMO support, would be of great interest in this regard.

### Strengths and Limitations

A major strength of the present study is the use of flow cytometry to investigate platelet function in ECMO patients with varying degrees of thrombocytopenia. Furthermore, by employing a model that describes platelet aggregation in healthy thrombocytopenic whole blood, we were able to evaluate platelet aggregation, measured by impedance aggregometry, relative to the platelet count.

The purpose of the present study was to contribute to further knowledge on the hemostatic changes occurring during ECMO support. Using two different methods, whole blood impedance aggregometry and flow cytometry, we aimed to elucidate different aspects of platelet function, however, other biomarkers and methods could have been employed: i.e., soluble levels of P-selectin or β-thromboglobulin, other surface-bound markers of platelet function (i.e., GPIbα, GPVI, CD40L), circulating platelet microparticles, circulating matrix metalloproteinases and platelet-leucocyte aggregates. Furthermore, this study is limited to the description of platelet count and function, not taking into account the wide interactions with leucocytes, nor blood-surface interactions throughout the circuit.

The results of the present study are confounded by the therapy some patients received while on ECMO support. In total, 57% of patients received platelet concentrates throughout the course of ECMO support (Table 2) (56). A time-dependent increase in platelet activation has been reported to occur during storage of platelet concentrates (57). Moreover, the response toward agonist stimulation has been shown to decrease during storage (56). Thus, our platelet function analyses are hampered by these exogenously provided platelets that have been activated during storage, and whose function is probably somewhat impaired. Additional, therapy during ECMO support included administration of antiplatelet drugs in nine patients and continuous administration of unfractionated heparin to the vast majority of patients (Table 1). Both antiplatelet agents and unfractionated heparin alter platelet function. Previous studies have reported unfractionated heparin to increase the number of activated platelets and sensitizes the platelets to agonist stimulation (58, 59). Thus, these therapies must be considered as confounders of the results in the present study.

Despite performing a sample size calculation and achieving the required number of included patients, the study population is rather small, limiting the possibilities for sub-group analysis or more advanced statistical analysis. An additional limitation is the lack of platelet function analyses on the entire study population prior to ECMO initiation. It remains unclear whether the observed platelet dysfunction can be associated with ECMO initiation, with the underlying disease state of the patients, or a combination of both. However, it is likely that initiating ECMO has a crucial impact on platelets, as platelet counts significantly decrease after ECMO initiation compared with before ECMO.

## Conclusions

We hypothesized that ECMO support would result in increased platelet function from day 1 to day 3 of support. Employing impedance aggregometry and flow cytometry, we found both impaired platelet aggregation and decreased platelet activation on day 1 of ECMO support compared with healthy controls. However, platelet aggregation was not impaired, when interpreted relative to the low platelet counts. Thus, our findings indicate that platelet aggregation during ECMO support is impaired due to a low number of platelets. Furthermore, levels of bound fibrinogen, on the surface of activated platelets in ECMO patients, were higher than in healthy controls. Together, these findings suggestively oppose that platelets are universally impaired during ECMO support. No marked difference in activation from day 1 to day 3 was seen during ECMO support.

## Data Availability

The datasets for this manuscript are not publicly available because of a small number of included participants. Requests to access the datasets should be directed to A-MH, am.hvas@dadlnet.dk.

## Ethics Statement

This study was approved as a quality assurance study by The Central Denmark Region Committees on Health and Research Ethics (enquiry 213-2016); therefore, according to Danish law, written informed consent was not required. The Danish Data Protection Agency approved the study (Ref. no. 1-16-02-712-17).

## Author Contributions

CB performed the laboratory analyses together with laboratory technicians Mai Stenulm Veirup and Vivi Bo Mogensen. CB extracted all clinical data from the patient medical records and took the lead in writing the manuscript. A-MH was responsible for the idea and scientific quality of the study. CB, A-MH, and AJ decided on which statistical analyses, tables and figures were to be included in the final manuscript. All authors contributed to a scientific discussion of the findings and provided critical revisions of the manuscript.

### Conflict of Interest Statement

The authors declare that the research was conducted in the absence of any commercial or financial relationships that could be construed as a potential conflict of interest.
