# Supplementary material for: Platelet Function During Extracorporeal Membrane Oxygenation in Adult Patients
Source: Front Cardiovasc Med. 2019 Aug 8;6:114. doi: 10.3389/fcvm.2019.00114 (PMC6694790; doi:10.3389/fcvm.2019.00114)

Supplementary Material

Supplementary Figure 1. A) Gating strategy for identification of platelets based on forward scatter intensity and surface expression of CD42b; **B)** Gating strategy for exclusion of platelet-platelet aggregates based on forward scatter intensity and forward scatter peak. **C)** Gating strategy of the negative control of samples with a platelet count >15 x 10^9/L; **D)** Gating strategy of the negative control of samples with a platelet count <15 x 10^9/L; Note the differences in the number of platelets assessed (Number, All) and in the percentage of positive platelets in the gates of the negative controls (%Gated, Bound Fibrinogen); **E)** Determination of platelet pre-activation in a sample with no added agonist; **F)** Expression of bound fibrinogen in a sample stimulated by collagen, applying the gate set using the negative control depicted in **C)**.

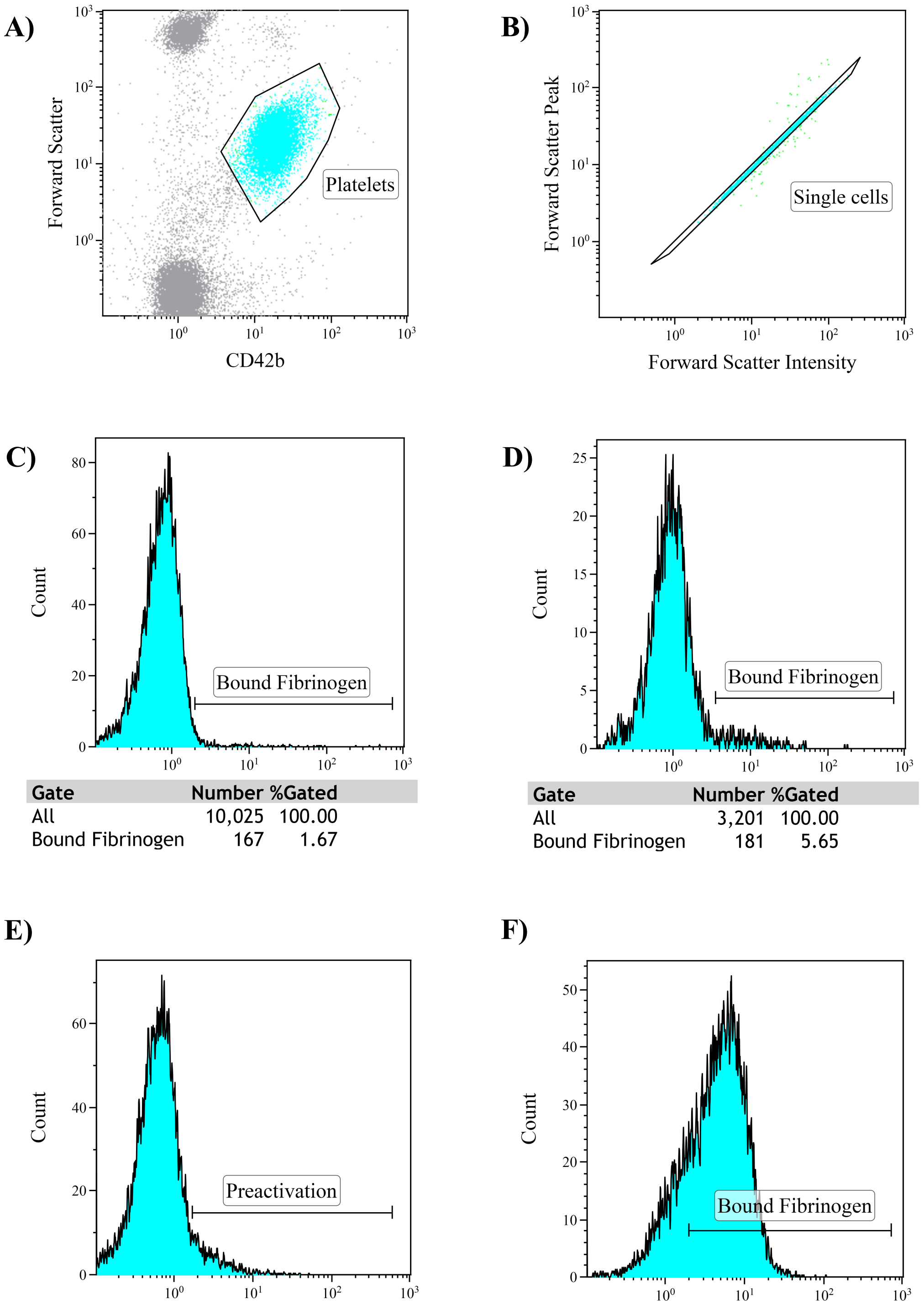

Supplement: Supplementary file 1 [file Image_1.pdf]
