# Supplementary material for: Platelet Function During Extracorporeal Membrane Oxygenation in Adult Patients
Source: Front Cardiovasc Med. 2019 Aug 8;6:114. doi: 10.3389/fcvm.2019.00114 (PMC6694790; doi:10.3389/fcvm.2019.00114)
Supplement: Supplementary file 2 [file Image_2.pdf]

Supplementary Material

Supplementary Figure 2. Illustrates the measured platelet aggregation during support with extracorporeal membrane oxygenation (ECMO) in relation to platelet aggregation in healthy whole blood at various platelet counts. Agonists used to stimulate platelet aggregation: Adenosine diphosphate (ADP) and thrombin receptor activating peptide-6 (TRAP). **The black line**: Represents the estimated platelet aggregation obtained by linear regression of data from healthy whole blood with varying degrees of thrombocytopenia (1). **The grey dashed line**: Represents the 95% prediction interval. An X-mark in the circle indicates that the patient has received platelet concentrate within 24 hours prior to blood sampling.

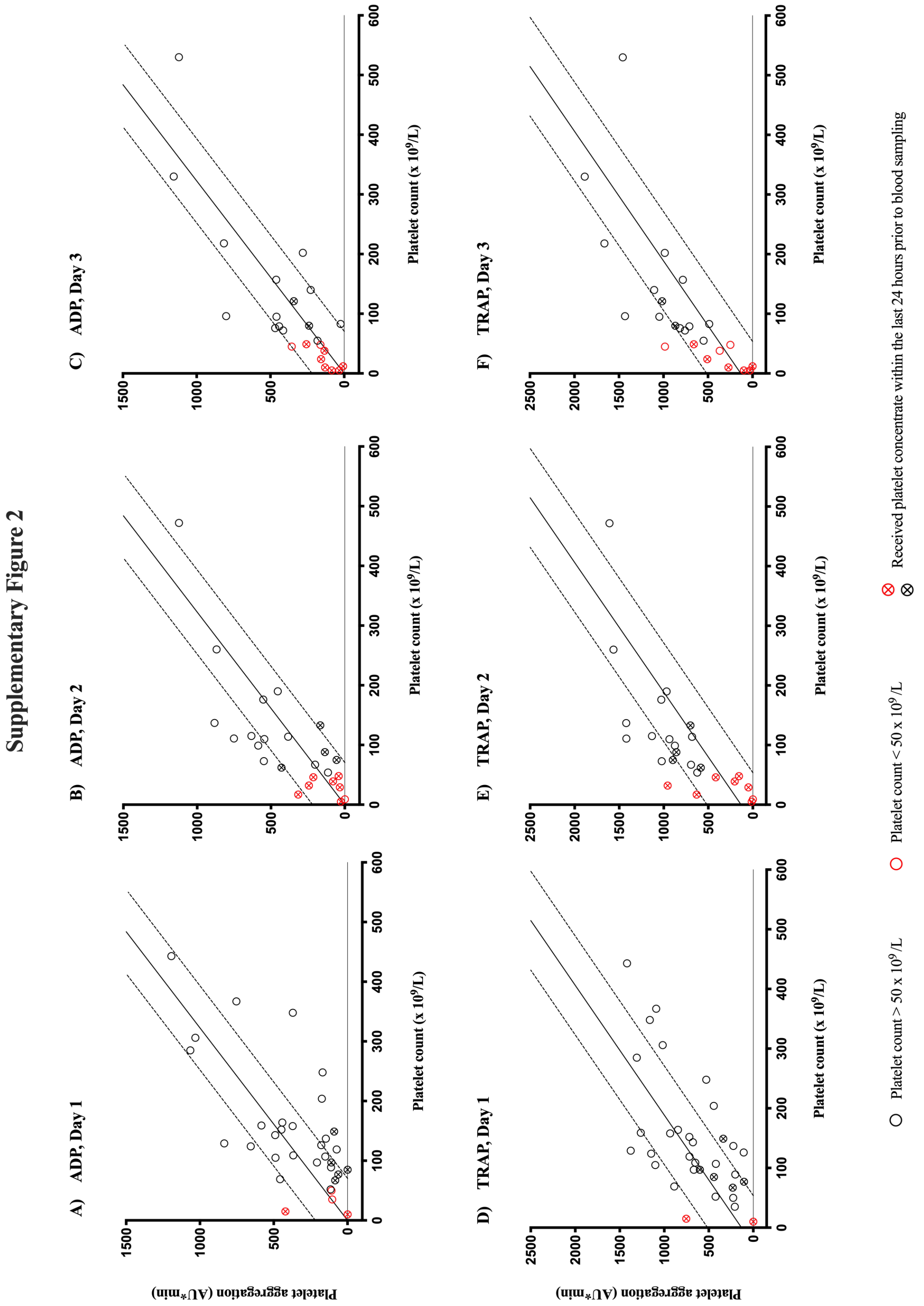

References

1. Skipper MT, Rubak P, Stentoft J, Hvas AM, Larsen OH. Evaluation of platelet function in thrombocytopenia. *Platelets* (2017):1-7. Epub 2017/04/15. doi: 10.1080/09537104.2017.1296566. PubMed PMID: 28409645.
